# Clinical overlap between functional neurological disorders and autism spectrum disorders: a preliminary study

**DOI:** 10.1192/j.eurpsy.2023.736

**Published:** 2023-07-19

**Authors:** D. Goeta, B. Demartini

**Affiliations:** 1 U.O. di Psichiatria, Presidio San Carlo; 2 U.O. di Psichiatria 52, Presidio San Paolo, ASST Santi Paolo e Carlo; 3 Aldo Ravelli Research Center for Neurotechnology and experimental brain therapeutics, University of Milan, milan, Italy

## Abstract

**Introduction:**

Functional neurological disorders (FNDs) and autism spectrum disorders (ASDs) share common features in terms of deficits in emotion regulation and recognition, sensory sensitivity, proprioception and interoception. Nevertheless, few studies have assesed teir overlap.

**Objectives:**

Aims of the present study were: (i) to assess the prevalence of autistic traits in a sample of adult patients with FNDs and (ii) to assess the prevalence of FNS in a sample of adult individuals with ASDs without intellectual disabilities; in this sample, we also evaluated the presence of a possible association between sensory sensitivity and FNS.

**Methods:**

We recruited 21 patients with FNDs, 30 individuals with ASDs without intellectual disabilities and 45 neurotypical adults (NA). Participants completed: the Autism Quotient (AQ); the Ritvo Autism Asperger Diagnostic Scale-Revised (RAADS-R); and a questionnaire assessing functional neurological symptoms (FNS). ASDs participants also completed the Sensory Perception Quotient-Short Form (SPQ-SF35), assessing sensory sensitivity.

**Results:**

In the FNDs sample, no patient scored above the clinical cut-off at the AQ and the 19% scored above the cut-off at the RAADS-R, a prevalence similar to the one we found in NA (15.6%; both p > 0.05). The 86.7% of participants with ASDs reported at least one FNS, a prevalence significantly higher than the NA one (35.6%, p < 0.001). In the ASDs sample, tactile hypersensitivity was found to be a risk factor for functional weakness (OR = 0.74, p = 0.033) and paraesthesia (OR = 0.753, p = 0.019).

**Conclusions:**

In conclusions, FNDs individuals did not present autistic traits more than NA, but ASDs individuals presented a higher number of FNSs than NA; this rate was associated with higher sensory sensitivity, especially in the touch domain.

**Disclosure of Interest:**

None Declared

